# Expanding the Search for Sperm Transmission Elements in the Mitochondrial Genomes of Bivalve Mollusks

**DOI:** 10.3390/genes12081211

**Published:** 2021-08-05

**Authors:** Donald T. Stewart, Brent M. Robicheau, Noor Youssef, Manuel A. Garrido-Ramos, Emily E. Chase, Sophie Breton

**Affiliations:** 1 Department of Biology, Acadia University, Wolfville, NS B4P 2R6, Canada; 2 Department of Biology, Dalhousie University, Halifax, NS B3H 4R2, Canada; brentrobicheau@acadiau.ca (B.M.R.); N.Youssef@dal.ca (N.Y.); 3 Departamento de Genética, Facultad de Ciencias, Universidad de Granada, 18071 Granada, Spain; mgarrido@ugr.es; 4 Institut Méditerranéen d’Océanologie, Aix-Marseille University, 13288 Marseille, France; emilychase@acadiau.ca; 5 Institut Hospitalo-Universitaire (IHU) Méditerranée Infection, 19-21 Boulevard Jean Moulin, 13005 Marseille, France; 6 Département de Sciences Biologiques, Université de Montréal, Montréal, QC H3C 3J7, Canada; s.breton@umontreal.ca

**Keywords:** mitochondrial DNA inheritance, sperm transmission elements, bivalves, DUI

## Abstract

Doubly uniparental inheritance (DUI) of mitochondrial DNA (mtDNA) in bivalve mollusks is one of the most notable departures from the paradigm of strict maternal inheritance of mtDNA among metazoans. Recently, work on the Mediterranean mussel *Mytilus galloprovincialis* suggested that a nucleotide motif in the control region of this species, known as the sperm transmission element (STE), helps protect male-transmitted mitochondria from destruction during spermatogenesis. Subsequent studies found similar, yet divergent, STE motifs in other marine mussels. Here, we extend the in silico search for mtDNA signatures resembling known STEs. This search is carried out for the large unassigned regions of 157 complete mitochondrial genomes from within the Mytiloida, Veneroida, Unionoida, and Ostreoida bivalve orders. Based on a sliding window approach, we present evidence that there are additional putative STE signatures in the large unassigned regions of several marine clams and freshwater mussels with DUI. We discuss the implications of this finding for interpreting the origin of doubly uniparental inheritance in ancestral bivalve mollusks, as well as potential future in vitro and in silico studies that could further refine our understanding of the early evolution of this unusual system of mtDNA inheritance.

## 1. Introduction

Over 100 species of bivalved mollusks are known to exhibit the unusual system of doubly uniparental inheritance (DUI) of mitochondrial DNA (mtDNA) [1]. DUI is an inheritance pattern characterized by two sex-associated lineages by which mtDNA is transmitted, a male lineage and a female lineage [2]. Typically, the male-transmitted mtDNA type (or M-type) is the exclusive type present in sperm whereas the female-transmitted type (or F-type) is the exclusive (or at least vastly predominant) type present in eggs and in somatic tissues of females [3]. The F-type is also the more abundant type in male somatic tissues, whereas the M-type is typically present in lower amounts [4]. DUI was first described in marine mussels (genus *Mytilus*) approximately 30 years ago [5,6]. In addition to marine mussels, DUI has been found in marine clams of the order Veneroida, freshwater mussels of the order Unionoida, and nutshells of the order Nuculanoida; however, the phylogenetic position of Nuculanoida (e.g., *Yoldia*) within or outside of the Protobranchia is controversial [1,7]. In a recent review of current research in the area of DUI, Stewart and colleagues [8] discussed the following topics: whether DUI evolved once or multiple origins, the challenges of annotating mtDNA genomes of DUI species, whether there is a link between the male- and female-transmitted mtDNA genomes and sex determination, what possible role(s) non-oxidative phosphorylation mitochondrial genes (i.e., open reading frames or ORFs) may play in freshwater mussels, and whether or not there are obvious evolutionary advantages of DUI, among other questions. One of the other points of discussion briefly considered was the possible function of conserved sequence motifs and sperm transmission elements (STEs) in the mitochondrial genomes of marine mussels. In this paper, we focus on this point by extending the analysis of Robicheau et al. [9] to search for conserved DNA sequence motifs that could represent possible sperm transmission elements as first described in Kyriakou et al. [10].

Within mitochondrial genomes, the search for molecular signals that may play a role in their sex-associated mode of transmission is facilitated by a particularly curious phenomenon best studied in marine mussels. As directly documented in several species within the blue mussel genus *Mytilus*, and inferred in several other members of the Mytilidae through genome and phylogenetic analyses, the male- and female-transmitted genomes in DUI positive taxa occasionally recombine (reviewed in [11]). While all manner of combinations of recombinant genomes are likely possibly, several known examples consist of recombinants composed of a mostly F-type genome (including the F-type protein coding genes, *12S* and *16S* ribosomal RNA genes, and most or all F-type transfer RNA genes) and an inserted control region from an M-type genome (e.g., [12]). Although there is variation in terms of exactly which additional adjacent portions of the male genome may also be recombined (e.g., [13]), this type of recombination event can result in a mostly female-type mitochondrial genome that is transmitted in a father-son pathway via sperm [14]. Such recombinant genomes have been referred to as recently masculinized (RM) genomes and the event is described as a “role-reversal,” which refers to the switch in sex-associated transmission for the majority of genes in these recombinant mitochondrial genomes [11]. An obvious conclusion to be drawn from the study of these recombinant genomes is that there could be a molecular signature (for e.g., a DNA motif) present in the M-type control region that facilitates its transmission from male parent to male offspring.

As summarized by Kyriakou et al. [10], there are three possible mechanisms that could result in the M-type dominating transmission in sperm while the F-type remains the predominant type found in somatic tissues of males: (i) only mitochondria from the male parent enter the primordial germ cells (PCGs) that will go on to populate the spermatogonia; (ii) mitochondria from both parents enter the PCGs but the M-type mitochondria experience more rapid replication and, in effect, outcompete the F-type mitochondria in this arena; and (iii) the F-type mitochondria are actively eliminated from the PCGs leaving only the M-type, which must also have entered the relevant blastomere in early development. Using electrophoretic mobility shift assays of gonad extracts from Mediterranean sea mussels (*Mytilus galloprovincialis*), Kyriakou et al. [10] provided evidence that there is a DNA sequence motif in the control region of the M-type that forms a DNA-protein complex. These authors also demonstrated that this DNA-protein interaction occurs in the perinuclear space within male gonad cells, and they proposed that these perinuclear mitochondria are protected from general degradation of cytosolic mitochondria that results in the destruction of the F-type mitochondria and their mtDNA genomes. Kyriakou and colleagues thus argued that the third scenario above, i.e., the destruction of the F-type and the preferential survival of the M-type, is the most likely of the three possible mechanisms underpinning the DUI system, at least in marine mussels [10].

Relatively little experimental or in silico work has been performed on mytilids over the past several years to follow up on the aforementioned work by Kyriakou et al. [10]. Robicheau et al. [9] used the STE signature of Kyriakou et al. [10], a 22–23 bp conserved DNA sequence string, to search for putative STE motifs in the highly divergent male and female-transmitted mitochondrial genomes of the horse mussel, *Modiolus modiolus* [15]. Overall, this work suggested that divergent, yet still recognizable, STEs may be found when less stringent search parameters are implemented during alignment procedures (e.g., by converting sequences to simply purines and pyrimidines). Indeed, a putative STE in the control region of the male mitochondrial genome of *M. modiolus* was identified using this more relaxed search approach [9]. Hence, the identification of homologous DNA sequence motifs that could be involved in the protection and (or) sequestration of the M-type mitochondrial genomes remains a feasible area for discovery despite the STEs’ rather short nucleotide signature.

In addition to the obvious relevance for understanding the evolution and maintenance of DUI in bivalve mollusks, studies of the molecular signatures involved in precisely controlling the nature and extent of mitochondrial heteroplasmy in animal cells have broad applicability in cell and molecular biology as noted by [16]. Mitochondrial heteroplasmy is implicated in human diseases, some quite severe, affecting a number of organ systems [17,18]. Teasing apart the mechanism(s) of the mitochondrial dynamics of the male- and female-transmitted mitochondrial genomes in bivalves could shed light on pathways of mitochondrial biogenesis (e.g., of the role of the nuclear-encoded gene PCG-1α [19,20] and mitophagy (e.g., the PINK1/Parkin axis that ultimately activates mitophagy of select mitochondria in animals [21]).

In this paper, we extend the search for putative STE signatures throughout the Bivalvia using a sliding window approach, reminiscent of the methodologies used in Robicheau et al. [9], to scan the large unassigned regions of available whole mitochondrial genomes for various bivalves. Collectively, our analyses provide evidence of putative STE motifs in the large unassigned regions of all three major lineages of bivalves that exhibit DUI, the Mytiloida, the Veneroida, and the Unionoida. Based on this finding, we then discuss strategies for further characterizing these molecular signatures/structures moving forward, as well as the broader implications of finding STE-like signatures throughout these three major lineages with respect to the evolution of doubly uniparental inheritance.

## 2. Materials and Methods

**Figure 1:**
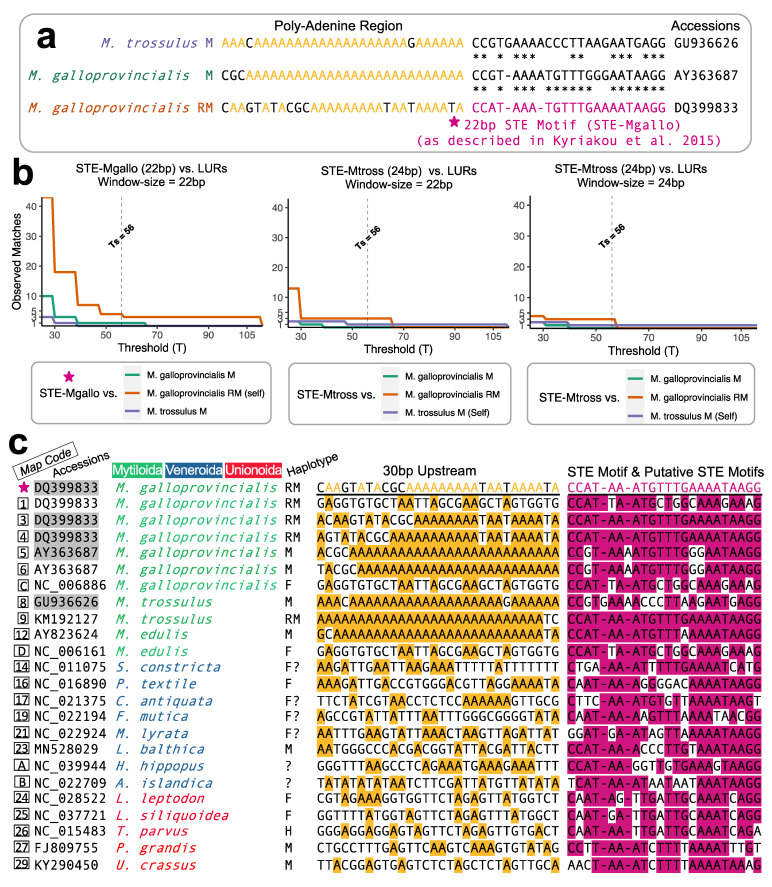
(**a**) Known sperm transmission elements (STEs) from Kyriakou et al. [10]. (**b**) Calibration of the sliding window procedure used to identify novel putative STE motifs. (**c**) Alignments of STE motifs from Kyriakou et al. [10] (accessions shown in shaded grey boxes) versus additional putative STE motifs identified in this study; the characterized STE motif from Kyriakou et al. [10] is marked with a ★. Nucleotides of the STE motif region were aligned using a MUSCLE algorithm [27], while the 30 bp upstream remains unaligned and simply shows adenine residues. H = Hermaphroditic mitotype, F = Female mitotype, M = Male mitotype, RM = Recently masculinized mitotype.

The definitive/known “STE motif” corresponds to the 22 bp nucleotide string reported by Kyriakou et al. [10] for an *M. galloprovincialis* RM-type that was shown to be functional via EMSAs: its sequence is 1bp–CCATAAATGTTTGAAAATAAGG–22bp. A diagram reiterating the homology between the definitive STE motif to other known STE signatures within closely related mytilids, as was originally proposed by Kyriakou et al. [10], is given in Figure 1a. As discussed elsewhere [8], the extremely short nucleotide length of the STE motif causes a significant challenge for identifying homologous STE signatures across Bivalvia. As an extreme example, the similarity between a previously described *M. trossulus* male-type STE versus the functional/known *M. galloprovincialis* RM-type STE was reported as ~63% (Figure 1a; also see [10] and the figures given therein). To overcome the issue of short sequence similarities, in this study, we made use of a sliding window approach implemented in Geneious Prime 2020.1.2 [22] to conduct a widespread search for putative STEs. The sliding window procedure in Geneious is based on EMBOSS dotmatcher [23]. From within Geneious, the sliding window parameters selected included a “high sensitivity/slow” algorithm and an exact scoring matrix that assumed no ambiguous matches (e.g., C vs. Y; [22]). The tile size for visualizations was set to 5000, and the sliding window size was set to the entire STE motif length of 22bp. The threshold selected for returning a positive match to the known STE motif was manually determined through calibration using known mytilid STE sequences along with their complementary large unassigned regions (LURs) from the sequences given in Kyriakou et al. [10]. We refer to this “threshold selected” value as Ts. The relationship between the sliding window Ts value and the number of observed matches (or alignment hits) during the sliding window procedure is shown in Figure 1b. The relationship is such that lower T values will lead to more spurious matches and potentially false positives, while higher T values will lead to fewer spurious matches but potential false negatives. Using the definitive STE motif (STE-Mgallo) and with prior knowledge that the quantity of STE alignment matches in *M. galloprovincialis* RM (DQ399833) should be *n* = 3, and in *M. galloprovincialis* M (AY363687) and *M. trossulus* M (GU936626) the STE quantity should be *n* = 1 each [10], a final threshold of Ts = 56 was selected. This threshold would allow for the observation of putative STEs in silico using the 22 bp STE motif, while also attempting to minimize false positive matches. We also found that the homologous 24 bp STE signature in the *M. trossulus* M mitotype (STE-Mtross) was unable to capture the definitive STE motif and its known duplications in *M. gallprovincialis* RM mitotype [10] when the sliding window was equivalent to the STE-Mtross motif length and above T = 57. Furthermore, although comparison to M. gallprovincialis M and M. trossulus M would suggest that a lower T value could have been selected for the known 22 bp STE motif (STE-Mgallo), we decided to select a more conservative T value under the assumption that it would be more informative to identify fewer potentially “true” STE matches, as opposed to capturing more numerous dissimilar STEs that may also have a higher likelihood of being false positives. Hence, we assume that our approach would still be unable to capture extremely ancient STE motifs that may fall below the threshold selected.

Given that STEs have already been discussed elsewhere for Mytiloida, we aimed to screen as many Veneroida and Unionoida bivalves as possible for putative STE motifs. However, to provide additional context we also screened twenty mitochondrial genomes for Ostreoida, as well as several mitochondrial genomes for Mytiloida. Complete mitochondrial genomes were retrieved from NCBI [24] using the Genome resource database therein (https://www.ncbi.nlm.nih.gov/genome/; accessed on 24 May 2021). Additional mitochondrial genomes (especially for M-types) were also retrieved from the nucleotide database of NCBI [24,25]. As best as possible, mitotype sex was inferred from NCBI sequence descriptors, for example, sometimes a DUI mitotype is specifically listed for a given sequence, while in other instances, tissue-type can suggest that a particular mt genome is an F-type based on the general principle that female-transmitted mtDNA is typically present in somatic tissues of both sexes [3,4]. Any mt genomes with an unresolved or uncertain transmission route based on the sequence descriptors are denoted by question marks. Overall, 157 mitochondrial genomes were retrieved for analysis (Supplementary Table S1). To facilitate the manual search of STE matches using the sliding window approach described above, STE motif searches were first limited to the large unassigned regions in each genome—these were manually extracted from complete mtDNA from within Geneious [22]. LURs are the positions most likely to house mitochondrial control regions [26] and are known to be implemented in the formation of recently masculinized mitotypes via mitochondrial recombination events [11]. The STE identified by Kyriakou et al. [10] was also found within a large unassigned control region. The positions of putative LURs were taken as the longest stretches of unannotated DNA between genes identified as either protein-coding (oxidative phosphorylation and ORF proteins) or ribosomal RNA. If the exact position of a DUI-related H-/F-/M-ORF was uncertain due to annotation inconsistencies (e.g., in *Unio pictorum*, the F-ORF was annotated as a “miscellaneous feature” rather than a gene), then it was not factored into the LUR boundaries. Transfer RNAs were not masked during the downstream sliding window analysis, nor were they excluded in the assessment of LURs. In total, 215 LURs were screened against the known 22 bp STE motif (STE-Mgallo). Any genome found to contain a putative STE motif within a LUR was then re-screened; for this second iteration, STE motifs were searched for within the entire circular mt genome. Final gene maps showing putative STE motif positions relative to other mitochondrial genes were generated in Geneious Prime 2020.1.2 [22], and then manually assembled into a composite display. In addition, the 30 bp upstream from putative STEs, as well as the putative STE motifs themselves (for those associated with LURs) were further retrieved (via Geneious) and inspected for sequence conservation. The 30 bp upstream was left unaligned and adenines highlighted; meanwhile, the putative STE nucleotides were aligned with the known STE motif (STE-Mgallo) using the MUSCLE algorithm [27] and then conserved nucleotide positions highlighted. The sequence similarity between each putative STE and the known 22 bp STE motif was further plotted from this alignment using the *ggplot2* [28] and *ggrepel* [29] packages in R version 4.0.3 [30] via RStudio version 1.3.1093 [31] (note *ggplot2* was also used for Figure 1b).

The distribution of putative STEs that could be attributed to LURs was further displayed on a bivalve phylogeny reconstructed in MEGAX version 10.1.8 [32,33] for the mitochondrial genomes screened. Since our objective was to visually display the putative STE motifs across the taxonomic orders investigated, as opposed to conducting a comprehensive Bivalvia evolutionary tree, we chose to use the simpler Neighbour-joining phylogenetic reconstruction method, as well as p-distances and pair-wise deletion [32,33]. The tree was also bootstrapped using 1000 replicates and made use of complete *co1* sequences that were retrieved from each of the complete mitochondrial genomes indicated earlier (accomplished through Geneious; [22]). Furthermore, said *co1* sequences were aligned via MUSCLE [27] and then trimmed to a conserved start/end nucleotide prior to constructing the phylogeny (the alignment and sequence trimming were both completed within MEGAX [32,33]). Onto this phylogeny, the major bivalve clades, as well as the lineages with putative STEs present, were shown through additional/manual annotation.

Given the small size of the known STE motif (22bp), the final stage of our analysis was to assess whether the putative STEs identified herein are likely to represent biologically significant matches between the known STE motif and LURs. This was accomplished by comparing the known STE motif to pseudo-sequences that were randomly generated. To this end, we generated 1000 random nucleotide sequences with lengths equivalent to the length of the LUR in the original analysis. The random sequences were generated using the empirical frequencies estimated from the original LUR (for either the forward or reverse DNA strand depending on putative STE orientation). For comparison, the analysis was repeated assuming equal nucleotide frequencies (i.e., 0.25 each). For each randomly generated sequence, we used a sliding window of 22 positions to identify potential STE motifs. A match was identified if the similarity between the window and the known STE was greater than or equal to the percent similarity observed for a newly identified putative STE motif (i.e., calculating potential false positive matches). Multiple matches could potentially be identified within a single sequence. We therefore report both the total number of false STE motifs found and the number of sequences with ≥1 false matches. This procedure allows us to estimate a pseudo *p*-value of the number of motif matches we would expect to find by random chance and in the absence of any duplication events; the estimate is calculated as the proportion of randomly generated sequences (out of 1000 replicates) with at least one motif match. This is similar to the ideas presented in [34]. Overall, the probabilities reported allow for a heuristic sense of whether the putative STE motifs identified during our analysis were likely to have originated from a mitochondrial region simply prone to displaying random matches to the known STE motif, or whether matches are more likely to have arisen from DUI relevant homologies. The statistical analyses above were carried out in Python [35] using the *NumPy* package [36] (see Supplemental Methods 1 for code).

## 3. Results and Discussion

The first extensive analysis of putative sperm transmission elements (STEs) in bivalve mollusks with the DUI system for mitochondrial DNA comes from the study by Kyriakou et al. [10], which focused on *Mytilus* spp. Accordingly, we used the 22 bp motif identified by those authors as a starting point to search for STEs in other bivalve mitochondrial genomes. As a critical first step, we needed to specify the search parameters to find a similarity threshold range that would minimize both false positive matches (i.e., searches that falsely identify non-homologous regions as matches due to low stringency) as well as minimizing false negative searches (i.e., searches with a stringency set so high that no or virtually no homologous regions were identified). As outlined earlier, a threshold of T = 56 for the known motif signature along with a sliding window length of 22 bp met these criteria (Figure 1a,b).

**Figure 2:**
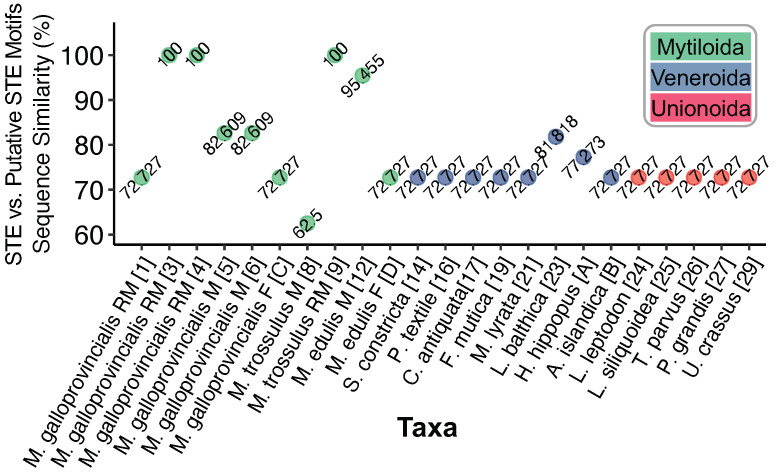
Degree of sequence similarity between STE-like signatures and the known 22 bp STE motif from Kyriakou et al. [10]. Nucleotides were aligned using MUSCLE [27].

Using the calibrated sliding window method above, we were able to identify a total of 23 STE-like signatures associated with LURs from the 157 mitochondrial genomes screened. Figure 1c shows the nucleotides identified as having homology to the known STE motif (pink). The 30 bp upstream from each signature (with adenines highlighted yellow) is also shown in Figure 1c. Out of the total LUR-associated putative STEs, four are previously known from Kyriakou et al. [10] (Map Codes 3, 4, 5, and 8) and one is the known STE motif (★), these are highlighted grey (Figure 1c). The STE signature from *M. trossulus* M (Map Code 8) was manually added to the analysis, as its relatively low sequence similarity makes it interesting for comparative purposes, yet it could not be identified using our search strategy (hence, this motif represents a true positive that falls below the threshold of detection). The degree of sequence similarity between the putative STE motifs of Figure 1c and the known STE is shown in Figure 2. Putative motifs range in sequence similarity from 62.5% (15/24bp) to 100% or 22/22bp.

**Figure 3:**
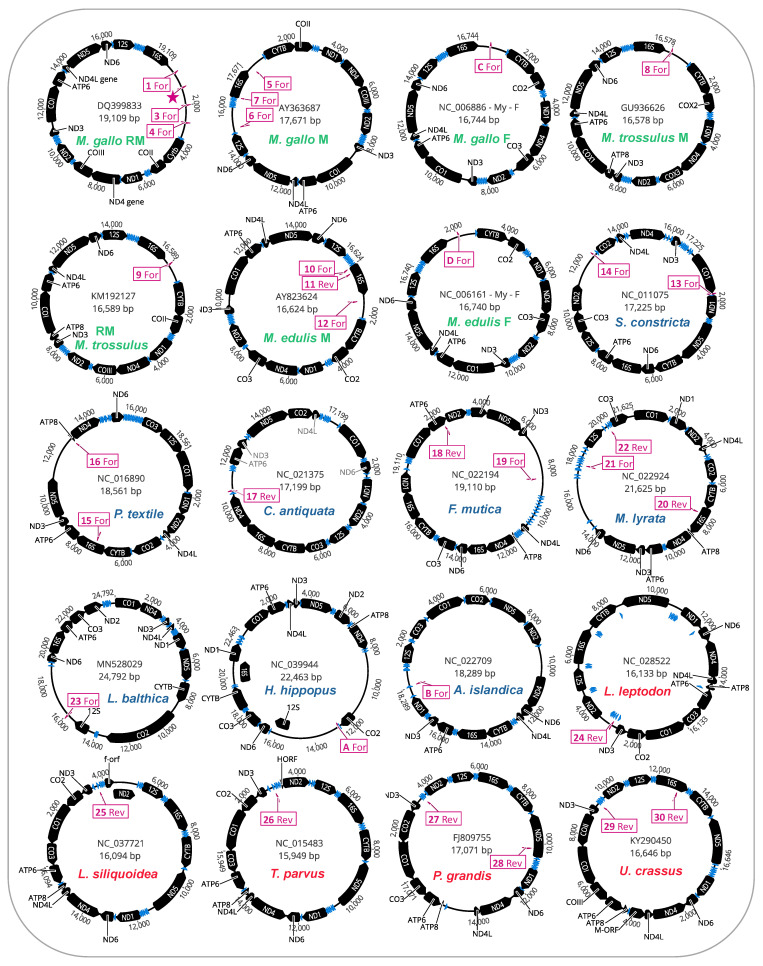
Mitochondrial genome maps for bivalve species exhibiting putative STE motifs localized to large unassigned regions in mtDNA. STE-like signatures are identified by map codes within boxes (these correspond to codes in Figure 1c) and can either be found on the Reverse (Rev) or Forward (For) mtDNA orientation. *M. gallo* refers to *M. galloprovincialis*.

We further investigated whether the mitochondrial genomes housing putative STE motifs in their LURs also displayed evidence of additional STE homology to other parts of their mitochondrial genomes (i.e., outside the LURs). The full genome maps for protein-coding genes, tRNAs, and putative STE motif signatures are given (Figure 3). As illustrated, for many of the genomes [12/20 mtDNAs] these motifs were only localized to the unassigned/control region (Figure 3). Interestingly, for the eight genomes with additional motifs outside the LURs, these were in many cases on the complementary DNA strand to the protein-coding or rRNA gene they were identified in and were usually associated with either *16S* (often), as well as *nd5*, *atp6*, or *co1* (less often; Figure 3). The localization of many putative STE motifs to LURs is interesting given that nearly two decades ago, Burzyński et al. [37], Zbawicka et al. [38] and Cao et al. [26] suggested that genetic signatures associated with DUI signaling were probably to be found in the non-coding portion of mtDNA as these unassigned regions or control regions, as they are variously called, are the mtDNA regions primarily associated with recombination events leading to the formation of masculinized mitochondrial genomes in the family Mytilidae.

**Figure 4:**
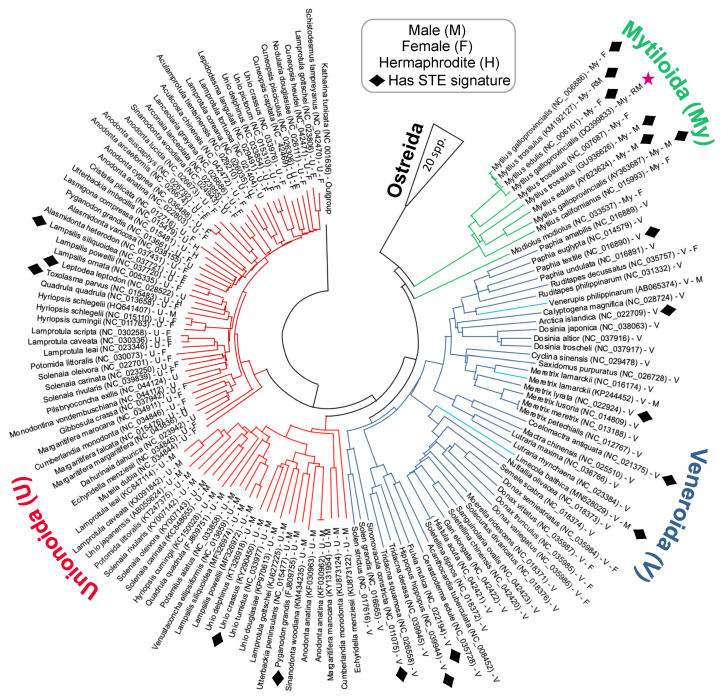
Taxonomic distribution of STE-like signatures discussed in this study. The phylogeny is a Neighbor-joining tree with 1000 bootstrap replicates reconstructed in MEGAX [32,33]. Full-length *co1* genes were aligned using MUSCLE [27] and trimmed to a conserved start/end nucleotide. Bootstrap values are shown in the expanded version of this phylogeny in Figure S1. ★ = Known STE motif from Kyriakou et al. [10]. The *Hyriopsis schlegelii* male sequence is likely an F-type genome derived from a male individual [43].

The phylogenetic distribution of bivalve mitotypes exhibiting putative STE signatures within their LURs is shown in the tree diagram presented in Figure 4. It is important to note that the Neighbour-joining tree in Figure 4 is based on the alignment of complete *co1* sequences. Although the tree is computationally quite simple, it nevertheless does an adequate job of representing the known broader evolutionary relationships previously indicated in the literature [1,39]. Overall, the phylogeny indicates that several distantly related clades from across the Unionida, Veneroida and Mytiloida have signatures similar to the known 22 bp STE motif (Figure 4). For those in Unionoida and Veneroida, signatures were found in thirteen species: three in DUI paternal genomes, two in DUI maternal genomes, one in an SMI hermaphroditic genome, and seven in genomes of unknown inheritance pattern for which we suppose are maternally-transmitted mtDNA (Figure 1). Despite our widespread finding of this signature, we did not observe their presence among taxa as a monophyletic group, nor were the signatures limited to male-transmitted genomes (Figure 4). For both trends, we must consider more closely that recombination between DUI mitotypes may obfuscate evolutionary patterns. As indicated earlier, recombination and masculinization of mtDNA genomes were demonstrated in the Mytilidae [13,40,41], and recombination and role-reversal have been events implicated in the other DUI positive bivalve orders [7,11]. Some of the male-transmitted mitochondrial genomes that were used during our analysis were known to be chimeric and, indeed, all M-types could be chimeric or derived from a chimeric ancestor. This means that the putative STE motifs are being mapped onto a genome tree rather than a gene tree. If we could confidently determine that recombination had occurred repeatedly and could reconstruct the recombination events throughout the hundreds of millions of years of bivalve evolutionary history, then we could determine whether the STE motif is monophyletic. Monophyly of the STE motifs across all bivalves would be strong evidence that the phenomenon evolved once. However, given the extremely long time periods involved, and the extremely small length of the putative STEs, reconstructing the evolutionary history of such events with greater confidence than we present here is highly unlikely. On a similar note, it is also possible that the putative STE motifs localized to F-type genomes could be the product of failed masculinization events (as suggested by the mt genomes analyzed in [13,42]), or alternatively, they may instead represent non-functional STEs that have yet to acquire enough nucleotide differences via mutation to appear dissimilar to the functional STE (as implied by sequences in Kyriakou et al. [10] for the *M. galloprovincialis* F-type genome). Accordingly, the presence of STE-like signatures in F-types does not allow us to definitively argue that DUI arose only once, in an ancestor to the Mytiloida, Unionoida, and Veneroida orders. If the DNA sequence motif of the STE was only found in sperm transmitted genomes and could be shown to be present in the Mytiloida, Unionoida, and Veneroida, then the most reasonable explanation would be that the phenomenon had evolved only once. While this is still the most parsimonious explanation, it is possible that the M-type sequestration mechanism evolved independently three or more times in the history of the Bivalvia. Analysis of additional complete sequences of M and F mitochondrial genomes in the future should help test hypotheses of putative cases of recombination and masculinization within particular bivalve orders, which should, in turn, shed more light on the roles of STEs and uncharacterized mitochondrial open reading frames (ORFs) in the origin and maintenance of DUI across the Bivalvia [8].

Despite these aforementioned challenges in understanding the evolutionary history of STEs, our search for putative STE motifs amongst a larger set of bivalve mitochondrial genomes has allowed for two major steps of progress in our understanding: (i) our analyses suggest that the STE signature may represent a unifying feature that is present within LURs from all three of the major lineages of bivalves exhibiting DUI (clams, freshwater mussels, and marine mussels) despite its origins still remaining unclear; and (ii) using the putative STEs identified herein, we are now positioned to design targeted electrophoretic mobility shift assays to detect DNA-protein complexes in Unionoida and Veneroida as was done for Mytiloida [10]. The most promising lineage currently appears to be *Limecola balthica* given its 81% similarity to the 22 bp STE motif (Figure 2). Moving forward, if the same DNA-protein complexes, and localization of perinuclear mitochondria can be demonstrated in the Unionoida as well as the Veneroida, this would be further strong evidence that the DUI phenomenon evolved once, but that there were subsequent role-reversal/recombination events in the ancestors to these two orders of bivalves as well.

Until relatively recent, sex-linked bivalve heteroplasmy was a central tenant of the DUI framework [44]. In DUI-positive species, males are typically heteroplasmic with the M-type dominating in sperm and male gonad, while the F-type dominates in all tissues of females as well as the somatic tissues of males. A recent analysis of the marine mussel *Semimytilus algosus*, which is hermaphroditic, has demonstrated that the sperm-producing gonad located on one half of the body contains one type of mtDNA while the egg-producing gonad located on the opposite side of the body contains another type of mtDNA [45]. This exception to the rule is still consistent with the hypothesis that some signal is present within the sperm-transmitted mitochondrial genome that is associated with a genetic mechanism that influences its fate (see [46], and particularly [47], for more advanced discussions on genetic signatures/models of DUI).

As a final component of our study, we further assessed whether STE matches are expected to represent true homology as opposed to false homology. False homology may arise as a product of random chance when the nucleotides in the short 22 bp motif are found stochastically, or if the frequency of nucleotides in the mtDNA being searched has a high overall similarity to the nucleotide frequency of the STE. The probabilities associated with identifying an STE match of comparable sequence similarity by random chance in the LURs examined are explored in Table 1; as the trends suggest, higher spurious matches are to be expected when the LUR size is larger and if the nucleotide frequencies in random sequence are equivalent to the LUR frequencies. There is also the possibility that a single random sequence may have more than one STE match; however, this is much rarer than individual sequences having at least one match (Table 1). Based on these analyses of 1000 replicates each, most of the novel LUR-associated STE matches except those for *F. mutica* and *M. lyrata* were significant (or near significant for Paphia textile, Arctica islandica, and Sinonovacula constricta). This is because out of 1000 searches there was a ≤5% probability (at *α* = 0.05) that at least one comparable (or better) match would be found by random chance. For less significant STE-like signatures the probability of observing a similar (or better) match at least once in 1000 random sequences was ~5–14% (Table 1). In contrast, we found that several putative STE motifs from Veneroida, and nearly all Unionoida matches, were very unlikely to have a similar (or better) STE signature by random chance; these probabilities were closer to ~0–1.5% (Table 1). Consequently, the random sequence tests suggest that the widespread occurrence of putative STE signatures across the three major DUI-exhibiting lineages is likely a true DUI-relevant feature.

In terms of future studies to further characterize the putative STEs presented herein, in addition to repeating the EMSA and DNA-protein complex analyses pioneered by Kyriakou et al. [10], further in silico analyses of the conserved DNA motifs of the putative STEs from M-type genomes from Veneroida and Unionoida should be focused on characterizing potential DNA bending properties that were hypothesized by Kyriakou and colleagues as crucial to the interaction of this STE motif with a dimerized protein complex. Additionally, in a follow-up paper [48], Kyriakou and colleagues showed that the homologous control region from the F-type mitochondrial genome of *M. galloprovincialis* was not capable of forming a stable protein structure comparable to the M-derived STE likely due in part to differences in the folding parameters. The ideas surrounding secondary structural folding may be even more relevant given that we did not observe any characteristic strings of adenine residues upstream from the putative STE motifs as was observed for various mytilids ([10]; Figure 1c).

## 4. Conclusions

Overall, our analyses have suggested that STE-like motifs appear to be widespread among several species of marine and freshwater mussels as well as clams. In agreement with previous findings in Kyriakou et al. [10], STEs within the mytilids remain an important feature of this group of bivalves. Based on the entire set of putative STEs identified herein, at present *Limecola balthica* appears a particularly promising study species for future functional experiments regarding STE motifs within the Veneroida. Although the analyses we provided in this study were rather extensive (screening many previously published mitochondrial bivalve genomes for STE-like signatures), our survey still understandably remains somewhat focused on maternally inherited mitochondrial genomes given that F genomes are often the ones reported by researchers. Consequently, as has been the case in past years, there is an ongoing need to sequence novel paternally inherited mitochondrial genomes. Finally, there of course remains the exciting prospect of characterizing the precise protein(s) that interact/bind with STE motif(s), and how/if STE signatures may fit into the framework of other DUI relevant bivalve mitochondrial features (e.g., smithRNAs discussed in [49]). Moving forward, future studies aimed at addressing these topics will be important for progressing our overall understanding of DUI in bivalves.

## Figures and Tables

**Table 1:** The probability of observing a comparable or better alignment match as those identified in the sliding window analysis. Probabilities are assessed based on generating 1000 random sequences of equal lengths to each Large Unassigned Region (LUR) under investigation. Reported are the total number of matches (Hits) found in the 1000 random sequences, the number of replicates (Seqs) where at least one or more matches were found, and the final probability of each LUR having a similar (or better) match as those found in our earlier analyses (/1000 replicates). Sequences were generated considering both the nucleotide frequency for each LUR ($\pi^{*}$) or assuming equal frequencies ($\pi_{i}=0.25,\;\mathrm{for}\;i\;\mathrm{equal}\;\mathrm{to}\;A,\;C,\;G,\;\mathrm{or}\;T)$. Frequencies are those of the forward (F) or reverse strand (R) depending on the orientation of the putative STE motif. Significant values (bold) are those where the number of replicates with one or more hits is ≤0.05.

Map Code (Strand)	ORDER Taxon	%	LUR bp	LUR $\pi^{*}=\langle \pi_{T},\;\pi_{C},\;\pi_{G},\;\pi_{A}\rangle$	*If* $\pi^{*}$			*If* $\pi_{i}\;=\;0.25$		
					Total Hits	Seqs. with ≥1 Hit	/1000	Total Hits	Seqs. with ≥1 Hit	/1000
	MYTILOIDA									
1 (F)	*Mytilus galloprovincialis* RM	72.7	3590	〈28.2, 15.8, 20.9, 35.0〉	85	81	0.081	14	14	**0.014**
6 (F)	*Mytilus galloprovincialis* M	77.3 ^a^	1529	〈30.1, 15.2, 18.9, 35.8〉	7	7	**0.007**	0	0	**0.000**
C (F)	*Mytilus galloprovincialis* F	72.7	1225	〈28.0, 14.6, 25.5, 31.9〉	11	11	**0.011**	3	3	**0.003**
9 (F)	*Mytilus trossulus* RM	100	1070	〈27.9, 15.3, 19.5, 37.3〉	0	0	**0.000**	0	0	**0.000**
12 (F)	*Mytilus edulis* M	95.5	993	〈28.9, 15.7, 19.4, 35.9〉	0	0	**0.000**	0	0	**0.000**
D (F)	*Mytilus edulis* F	72.7	1226	〈28.1, 14.3, 25.1, 32.5〉	17	17	**0.017**	4	4	**0.004**
	VENERIDA									
14 (F)	*Sinonovacula constricta*	72.7	1602	〈33.1, 8.40, 24.7, 33.8〉	36	35	**0.035**	6	6	**0.006**
16 (F)	*Paphia textile*	72.7	1986	〈26.8, 11.9, 25.4, 35.9〉	53	51	0.051	9	9	**0.009**
17 (R)	*Coelomactra antiquata*	72.7	1285	〈31.4, 27.6, 13.3, 27.6〉	7	7	**0.007**	4	4	**0.004**
19 (F)	*Fulvia mutica*	72.7	4368	〈30.7, 12.3, 22.1, 34.9〉	108	106	0.106	9	9	**0.009**
21 (F)	*Meretrix lyrata*	72.7	4620	〈41.5, 5.80, 20.8, 31.9〉	142	137	0.137	12	12	**0.012**
23 (F)	*Limecola balthica*	81.8	3968	〈35.8, 10.6, 20.9, 32.7〉	0	0	**0.000**	0	0	**0.000**
A (F)	*Hippopus hippopus*	77.3	3027	〈33.4, 12.1, 25.7, 28.8〉	4	4	**0.004**	1	1	**0.001**
B (F)	*Arctica islandica*	72.7	1497	〈37.4, 10.2, 17.4, 35.0〉	52	52	0.052	6	6	**0.006**
	UNIONIDA									
24 (R)	*Leptodea leptodon*	72.7	830	〈37.5, 8.40, 26.5, 27.6〉	16	15	**0.015**	3	3	**0.003**
25 (R)	*Lampsilis siliquoidea*	72.7	575	〈34.8, 11.1, 23.8, 30.3〉	10	10	**0.010**	4	4	**0.004**
26 (R)	*Toxolasma parvus*	72.7	647	〈32.0, 10.8, 25.8, 31.4〉	13	13	**0.013**	0	0	**0.000**
27 (R)	*Pyganodon grandis*	72.7	575	〈33.7, 11.7, 19.8, 34.8〉	9	9	**0.009**	2	2	**0.002**
29 (R)	*Unio crassus*	72.7	557	〈31.2, 13.5, 20.3, 35.0〉	12	12	**0.012**	0	0	**0.000**

^a^ STE motif is likely 23bp, however, the 22 bp identified by the sliding window was a 17/22 bp match.

## Data Availability

The datasets generated in this study represent a form of meta-analysis; original data sources are referenced within the manuscript itself and (or) listed as sequence accession codes provided in Table S1.

## Supplementary Materials

The following are available online at https://www.mdpi.com/article/10.3390/genes12081211/s1, Figure S1: Expanded view of Figure 4, Table S1: List of all mitochondrial genomes used in this study, Supplemental Methods 1: Code for random sequence tests.
