# Supplementary material for: Expanding the Search for Sperm Transmission Elements in the Mitochondrial Genomes of Bivalve Mollusks
Source: Genes (Basel). 2021 Aug 5;12(8):1211. doi: 10.3390/genes12081211 (PMC8394068; doi:10.3390/genes12081211)
Supplement: Supplementary file 1 [file genes-12-01211-s001.zip › genes-1294084-supplementary.pdf]

Supplemental Information for:

Expanding the search for sperm transmission elements in the mitochondrial genomes of bivalve mollusks

Donald T Stewart [1, a,*], Brent M Robicheau [2, a], Noor Youssef [2], Manuel A Garrido-Ramos [3], Emily E Chase [4] and Sophie Breton [5]

Table S1. List of all mitochondrial genomes used in this study. Accession codes can be searched in NCBI [23] for additional information on original publication sources, sequence author(s), etc. Taxonomic name listed is as found in the original GenBank report; some taxonomic names are under debate and may have changed since these sequences were first reported.

Taxonomy from Sequence Definition	Accessions	Taxonomic Synonym Given at NCBI
Acanthocardia tuberculata	NC_008452	—
Aculamprotula tientsinensis	NC_029210	—
Acuticosta chinensis	NC_042472	—
Alasmidonta heterodon	NC_037431	—
Alasmidonta varicosa	NC_038155	—
Anodonta anatina	NC_022803	—
Anodonta anatina	KF030962	—
Anodonta anatina	KF030963	—
Anodonta arcaeformis	NC_026674	—
Anodonta cygnea	NC_036488	—
Anodonta euscaphys	NC_026792	*Anemina euscaphys*
Anodonta lucida	NC_026673	—
Arconaia lanceolata	NC_023955	*Lanceolaria lanceolata*
Arctica islandica	NC_022709	—
Calyptogena magnifica	NC_028724	—
Cerastoderma edule	NC_035728	—
Coelomactra antiquata	NC_021375	*Mactra antiquata*
Crassostrea angulata	NC_012648	—
Crassostrea ariakensis	NC_012650	—
Crassostrea belcheri	NC_037851	—
Crassostrea gasar	NC_027653	—
Crassostrea gigas	NC_001276	—
Crassostrea hongkongensis	NC_011518	—
Crassostrea iredalei	NC_013997	—
Crassostrea nippona	NC_015248	—
Crassostrea sikamea	NC_012649	—
Crassostrea sp.	NC_018763	—
Crassostrea virginica	NC_007175	—
Cristaria plicata	NC_012716	—
Cumberlandia monodonta	KU873124	—

Cumberlandia monodonta	NC_034846	—
Cuneopsis capitata	NC_042469	*Cuneopsis capitatus*
Cuneopsis heudei	NC_042471	—
Cuneopsis pisciculus	NC_026306	—
Cyclina sinensis	NC_029478	—
Dahurinaia dahurica	NC_023942	*Margaritifera dahurica*
Donax semiestriatus	NC_035984	—
Donax trunculus	NC_035985	—
Donax variegatus	NC_035986	—
Donax vittatus	NC_035987	—
Dosinia altior	NC_037916	—
Dosinia japonica	NC_038063	—
Dosinia troscheli	NC_037917	—
Echyridella menziesii	KU873122	—
Echyridella menziesii	NC_034845	—
Fulvia mutica	NC_022194	—
Gari elongata	NC_042422	—
Gibbosula crassa	NC_037942	—
Hiatula acuta	NC_042421	—
Hippopus hippopus	NC_039944	—
Hyriopsis cumingii	NC_011763	—
Hyriopsis schlegelii	NC_015110	*Sinohyriopsis schlegelii*
Hyriopsis schlegelii	HQ641407	*Sinohyriopsis schlegelii*
Katharina tunicata (Outgroup for tree)	NC_001636	—
Lamprotula caveata	KX091842	—
Lamprotula caveata	NC_030336	—
Lamprotula coreana	NC_026035	—
Lamprotula gottschei	KJ627225	—
Lamprotula gottschei	NC_023806	—
Lamprotula leai	NC_023346	*Lamprotula leaii*
Lamprotula leai	KC847114	*Lamprotula leaii*
Lamprotula scripta	NC_030258	—
Lamprotula tortuosa	NC_021404	—
Lampsilis ornata	NC_005335	—
Lampsilis powellii	NC_037720	—
Lampsilis powellii	MF326972	—
Lampsilis siliquoidea	MF326974	—
Lampsilis siliquoidea	NC_037721	—
Lanceolaria grayana	NC_026686	*Lanceolaria grayii*
Lasmigona compressa	NC_015481	—
Lepidodesma languilati	NC_029491	—
Leptodea leptodon	NC_028522	*Potamilus leptodon*
Lutraria maxima	NC_036766	—

Lutraria rhynchaena	NC_023384	—
Mactra chinensis	NC_025510	—
Margaritifera falcata	NC_015476	—
Margaritifera margaritifera	NC_043836	—
Margaritifera marocana	NC_034911	*Pseudunio marocanus*
Margaritifera marocana	KY131954	*Pseudunio marocanus*
Meretrix lamarckii	NC_016174	—
Meretrix lamarckii	KP244452	—
Meretrix lusoria	NC_014809	—
Meretrix lyrata	NC_022924	—
Meretrix meretrix	NC_013188	—
Meretrix petechialis	NC_012767	—
Modiolus modiolus	NC_033537	—
Moerella iridescens	NC_018371	*Iridona iridescens*
Monodontina vondembuschiana	NC_044112	—
Mutela dubia	NC_034844	—
Mytilus californianus	NC_015993	—
Mytilus edulis	AY823624	—
Mytilus edulis	NC_006161	—
Mytilus galloprovincialis	AY363687	—
Mytilus galloprovincialis	NC_006886	—
Mytilus galloprovincialis	DQ399833	—
Mytilus trossulus	GU936626	—
Mytilus trossulus	KM192127	—
Mytilus trossulus	NC_007687	—
Nodularia douglasiae	NC_026111	—
Nuttallia olivacea	NC_018373	—
Ostrea denselamellosa	NC_015231	—
Ostrea edulis	NC_016180	—
Ostrea lurida	NC_022688	—
Paphia amabilis	NC_016889	—
Paphia euglypta	NC_014579	—
Paphia textile	NC_016890	*Paratapes textilis*
Paphia undulata	NC_016891	*Paratapes undulatus*
Pilsbryoconcha exilis	NC_044124	—
Potamilus alatus	NC_033858	—
Potomida littoralis	NC_030073	—
Potomida littoralis	KT247375	—
Pyganodon grandis	FJ809755	—
Pyganodon grandis	NC_013661	—
Quadrula quadrula	FJ809751	—
Quadrula quadrula	NC_013658	—
Ruditapes decussatus	NC_035757	—

Ruditapes philippinarum	NC_031332	—
Saccostrea cucullata	NC_027724	—
Saccostrea echinata	NC_036478	—
Saccostrea glomerata	NC_036483	—
Saccostrea kegaki	NC_030533	—
Saccostrea mordax	NC_013998	—
Saccostrea mytiloides	NC_036479	—
Sanguinolaria ovalis	NC_042423	—
Saxidomus purpuratus	NC_026728	*Saxidomus purpurata*
Schistodesmus lampreyanus	NC_042470	*Schistodesmus lampreyanus*
Semele scabra	NC_018374	—
Sinanodonta woodiana	KM434235	—
Sinanodonta woodiana	NC_024943	—
Sinonovacula constricta	NC_011075	—
Solecurtus divaricatus	NC_018376	—
Solen grandis	NC_016665	—
Solen strictus	NC_017616	—
Solenaia carinata	NC_023250	—
Solenaia carinata	KC848655	—
Solenaia oleivora	NC_022701	—
Solenaia oleivora	KY007143	—
Solenaia rivularis	NC_039839	*Parvasolenaia rivularis*
Solenaia rivularis	KY007142	*Parvasolenaia rivularis*
Soletellina chinensis	NC_042420	*Hiatula chinensis*
Soletellina diphos	NC_018372	*Hiatula diphos*
Toxolasma parvus	NC_015483	*Toxolasma parvum*
Tridacna derasa	NC_039945	—
Tridacna squamosa	NC_026558	—
Unio crassus	KY290450	—
Unio crassus	NC_033976	—
Unio delphinus	NC_033854	—
Unio delphinus	KT326918	—
Unio douglasiae	KP970613	*Nodularia douglasiae*
Unio japanensis	AB055624	*Pronodularia japanensis*
Unio pictorum	NC_015310	—
Unio tumidus	NC_033977	—
Hyriopsis cumingii	KC150028	—
Limecola balthica	MN528029	—
Utterbackia imbecillis	NC_015479	—
Utterbackia peninsularis	NC_015477	—
Venerupis philippinarum	AB065374	*Ruditapes philippinarum*
Venustaconcha ellipsiformis	NC_013659	—

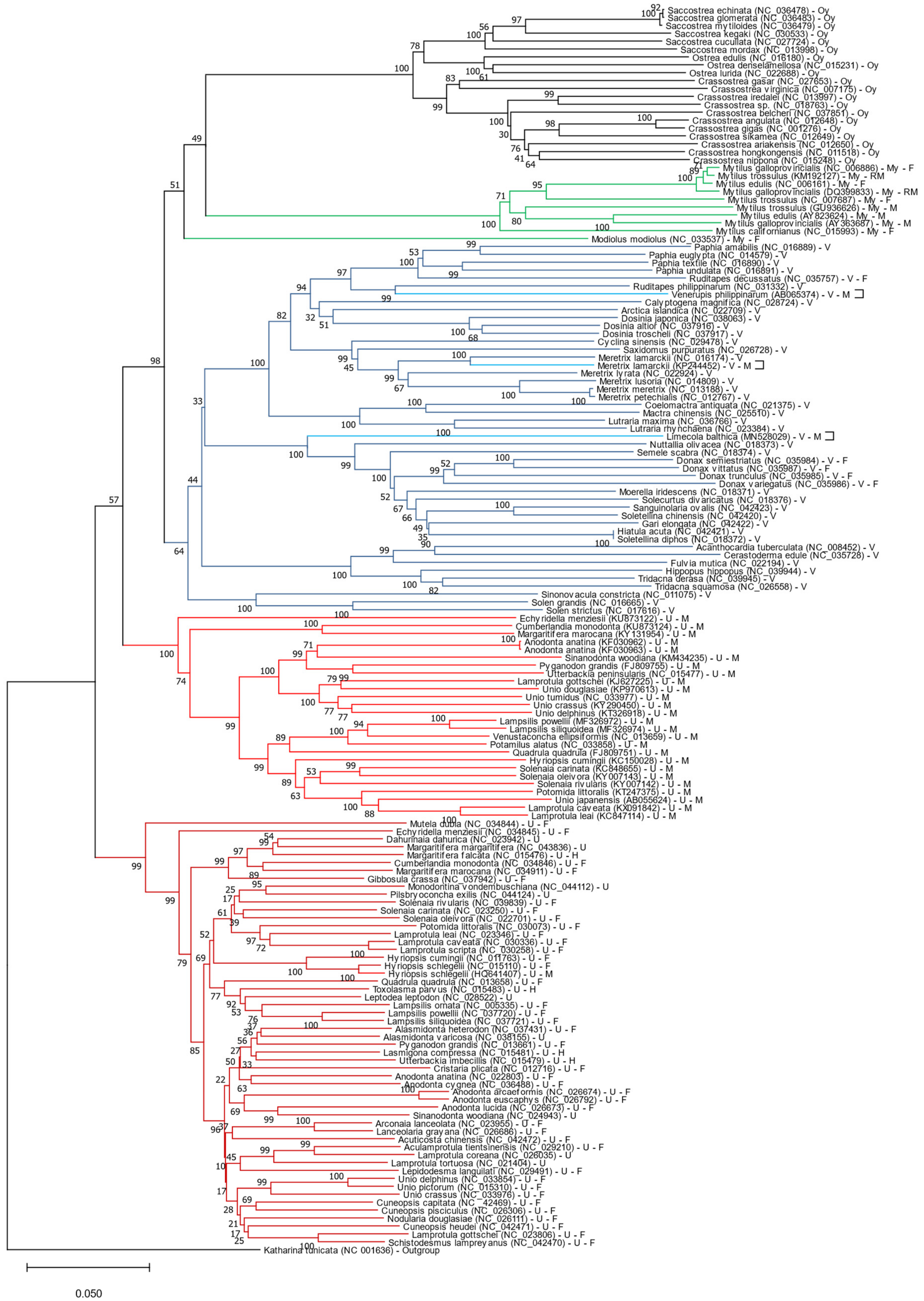

Figure S1. Expanded view of Figure 4. Image shows bootstrap values and full view of Ostreoida clade (top clade shown in black).

Supplemental Methods 1. Code for random sequence tests. The following text can be saved as a .py file and then ran via Python [34] to conduct the statistical tests used herein. Nuc_motif = nucleotide signature of 22bp STE motif; length = LUR lengths; frequencies = nucleotide frequencies of LURs in the order T, C, G,

and A; threshold = amount of minimum similarity (bp) required for a match.

```
#!/usr/bin/env python3
# -*- coding: utf-8 -*-
"""
Created on Tue Aug  6 11:48:36 2019
Modified on Fri May 21 2021
@author: Noor Youssef

Identifying STE motifs in random sequences
"""
import numpy as np

## Parameters to change ##
nuc_motif = "CCATAAATGTTTGAAAATAAGG"

#specify sequence length
length = {"MgaRM" : 3590, "MgaSM": 1529, "MgaF": 1225, "MtrRM": 1070, "MedM": 993, "MedF": 1226,
"Sco": 1602, "Pte": 1986,
         "Can": 1285, "Fmu": 4368, "Mly": 4620, "Lba": 3968, "Hhi": 3027, "Ais": 1497, "Lle":
830, "Lsi": 575, "Tpa": 647, "Pgr": 575, "Ucr": 557}

#specify frequencies pi_T, pi_C, pi_G, pi_A
frequencies = { "MgaRM"  :[28.2,15.8,20.9,35.0],
                 "MgaSM" :[30.1,15.2,18.9,35.8],
                 "MgaF" :[28.0,14.6,25.5,31.9],
                 "MtrRM" :[27.9,15.3,19.5,37.3],
                 "MedM" :[28.9,15.7,19.4,35.9],
                 "MedF" :[28.1,14.3,25.1,32.5],
                 "Sco" :[33.1,8.40,24.7,33.8],
                 "Pte" :[26.8,11.9,25.4,35.9],
                 "Can" :[31.4,27.6,13.3,27.6],
                 "Fmu" :[30.7,12.3,22.1,34.9],
                 "Mly" :[41.5,5.80,20.8,31.9],
                 "Lba" :[35.8,10.6,20.9,32.7],
                 "Hhi" :[33.4,12.1,25.7,28.8],
                 "Ais" :[37.4,10.2,17.4,35.0],
                 "Lle" :[37.5,8.40,26.5,27.6],
                 "Lsi" :[34.8,11.1,23.8,30.3],
                 "Tpa" :[32.0,10.8,25.8,31.4],
                 "Pgr" :[33.7,11.7,19.8,34.8],
                 "Ucr" :[31.2,13.5,20.3,35.0]}

# specify threshold
threshold = {"MgaRM" :16,
             "MgaSM" :17,
             "MgaF" :16,
             "MtrRM" :22,
             "MedM" :21,
             "MedF" :16,
             "Sco" :16,
             "Pte" :16,
             "Can" :16,
             "Fmu" :16,
             "Mly" :16,
             "Lba" :18,
             "Hhi" :17,
             "Ais" :16,
             "Lle" :16,
             "Lsi" :16,
             "Tpa" :16,
             "Pgr" :16,
             "Ucr" :16}
##########################################################################################
nucleotide_converter = {"T":0, "C":1, "G":2, "A":3}
motif =  [nucleotide_converter[i] for i in nuc_motif]

def count_num_of_hits(seq, motif, threshold):
    '''
```

```
    Counts the number of times (i.e., hits) a given motif is observed in a sequence given a
threshold (e.g, 16 nucleotide matches)

    seq: full sequence
    motif: segement we are looking for
    threshold: the number of positions that must match
    '''
    number_hits = 0

    # sliding window over sequence
    for i in range(len(seq) - len(motif) + 1):
        window = seq[i:i+len(motif)]

        #count the number of similar nucleotides between motif and window
        similarity = sum([1 for x in range(len(motif)) if window[x] == motif[x]])

        #if similarity greater than threshold, count as hit
        if similarity >= threshold:
            number_hits += 1
    return(number_hits)


# for each organism extract sequence length, nucleotide frequencies, and threshold
for org in length.keys():
    seq_length = length[org]
    freq       = frequencies[org]
    thresh     = threshold[org]

    num_seqs_with_motif = []; num_seqs_unifrom_with_motif = []
    for trial in range(1000):

       #generate a random sequence generated from the empirical nucleotide frequency
        seq = np.random.choice(4, seq_length, p= [x/sum(freq) for x in freq])
        seq_unifrom = np.random.choice(4, seq_length, p= [0.25, 0.25, 0.25, 0.25])

        #count the number of times motif was present in the random sequence
        number_hits_per_seq = count_num_of_hits(seq, motif, thresh)
        number_hits_per_seq_unifrom = count_num_of_hits(seq_unifrom, motif, thresh)

        if number_hits_per_seq > 0:
            num_seqs_with_motif.append(number_hits_per_seq)

        if number_hits_per_seq_unifrom > 0:
            num_seqs_unifrom_with_motif.append(number_hits_per_seq_unifrom)

    print("Sequences generated from empirical frequencies")
    print(org, "Total number of hits", sum(num_seqs_with_motif), "Number of sequences with at-
least one hit", len(num_seqs_with_motif))

    print("Sequences generated from uniform frequencies")
    print(org, "Total number of hits", sum(num_seqs_unifrom_with_motif), "Number of sequences
with atleast one hit", len(num_seqs_unifrom_with_motif))
```
